# Robust Detection of Abandoned Object for Smart Video Surveillance in Illumination Changes

**DOI:** 10.3390/s19235114

**Published:** 2019-11-22

**Authors:** Hyeseung Park, Seungchul Park, Youngbok Joo

**Affiliations:** School of Computer Science and Engineering, Korea University of Technology and Education, 1600 Chungjeol-ro, Byeongcheon-myeon, Dongnam-gu, Cheonan-si, Chungcheongnam-do, Korea; hs2000park@koreatech.ac.kr (H.P.); ybjoo@koreatech.ac.kr (Y.J.)

**Keywords:** abandoned object detection, PETS2006, ABODA, occlusion, illumination change, smart video surveillance, unattended object detection

## Abstract

Most existing abandoned object detection algorithms use foreground information generated from background models. Detection using the background subtraction technique performs well under normal circumstances. However, it has a significant problem where the foreground information is gradually absorbed into the background as time passes and disappears, making it very vulnerable to sudden illumination changes that increase the false alarm rate. This paper presents an algorithm for detecting abandoned objects using a dual background model, which is robust even in illumination changes as well as other complex circumstances like occlusion, long-term abandonment, and owner re-attendance. The proposed algorithm can adapt quickly to various illumination changes. And also, it can precisely track the target objects to determine whether it is abandoned regardless of the existence of foreground information and the effect from the illumination changes, thanks to the largest-contour-based presence authentication mechanism proposed in this paper. For performance evaluation, we trialed the algorithm with the PETS2006, ABODA datasets as well as our dataset, especially to demonstrate its robustness in various illumination changes.

## 1. Introduction

In the last few years, there has been a growing interest in intelligent video surveillance systems that automatically detect certain events such as intrusion, loitering, abandonment, and fire without the need for constant observation of humans. Among them, many pieces of research for detecting abandoned objects have been conducted, covering illegal dumping, illegally parked vehicles, or suspicious objects related to terrorism [1,2,3,4].

An abandoned object is an object left by its owner and not re-attended within a pre-defined time [5]. Most existing abandoned object detection algorithms use foreground information generated from background models. The background subtraction technique is one of the effective ways to extract foreground information from images. One of the problems is that the foreground is gradually absorbed into the background over time, which can be an obstacle for algorithms highly dependent on the foreground information to detect and track the target objects. Another problem is that it is very vulnerable to illumination changes. Illumination changes can occur in various situations, including a light being turned on/off and cloud covering the sun. When the changes happen, the whole image can be affected. The affected parts of the image are then exposed as the new foregrounds, which could last for a long time in the following consecutive images depending on the learning rate of the background model. It can significantly increase the false alarm rate, which leads to the poor performance of a surveillance system. Therefore, a technique quickly detecting and adapting to illumination changes while continuing to track the objects is necessary.

To detect abandoned objects, we use a dual background model consisting of two background models with different learning rates because it can efficiently extract stationary objects with relatively less computation cost [6]. Besides, we additionally deploy a rapid adaptation mechanism to handle illumination changes and the presence authentication mechanism to determine the next or final state of a candidate stationary object. The proposed algorithm detects the occurrence of an illumination change when detecting a huge foreground blob from the model. If an illumination change is detected, the algorithm executes the adaptation mechanism. It immediately increases the learning rates of both models equally to make them adapt quickly to the change at the same time, which means that they absorb all of the affected parts into the background. We then need to know whether the adaptation perfectly finishes. The termination of the adjustment can be determined by checking that there is no blob extracted in the difference image of short-term and long-term foreground images, which means that the foregrounds of both models become completely identical after discarding the previous background history. In other words, they perfectly adapt to the illumination change. After absorbing all affected parts into the background, each learning rate is returned to its original value.

The illumination change handling technique introduced above has the advantage of quickly detecting and adapting to the changes, which prevents the algorithm from extracting wrong candidate stationary objects. However, it also has a limitation where existing meaningful foreground information generated before the changes can disappear after adaptation. To solve this problem, we suggest the template registration and presence authentication. Before that, we first need to select a candidate stationary object meeting some prerequisites, such as stability. When a candidate stationary object is selected, its attributes like position, size, and the largest contour of the object image are registered together. In this paper, we call this set of attributes the template of the candidate stationary object. Afterward, the foreground of the registered object is no longer needed to determine the state of the object. The proposed algorithm only requires the template, which makes it possible for a surveillance system to track an object for long periods, regardless of the problem where the foreground absorbs into the background over time. It does not perform any visual analysis for tracking until the pre-defined *n* seconds have passed since the candidate stationary object was unattended by its owner. When the timer for abandonment determination expires, the algorithm verifies the existence of the target object in the current video frame to determine whether it is abandoned. We call this the presence authentication, which is performed by comparing the largest contour of the candidate stationary object in the template and that of the current object image. The reason for using the largest contour, not others such as the whole contours or histogram, is because it is more robust to illumination changes according to our experiments. The result of the comparison determines the next or final state of the object. So, the proposed algorithm can work robustly against illumination changes as well as other complex issues such as occlusion and long-term abandoned object tracking. This paper mainly focuses on the robust detection of abandoned objects in illumination changes.

## 2. Related Works

Most of the existing studies for abandoned object detection analyze foreground information generated from one or more background models to distinguish between stationary and dynamically moving objects and track the stationary objects for a certain period to determine whether they are abandoned. Q. Fan et al. [7] lower the false alarm rate by modeling a temporarily static object, such as a car that stops for a while and then moves again, using a single background model and finite state machine (FSM). Their study also considered the healed object which has already absorbed into the background, but the illumination change issue is not covered. On the other hand, Y. Tian et al. [8,9] use a single background model consisting of three Gaussian mixture models. Also, intensity and texture information are integrated to remove shadows and to be robust to illumination changes. The assumption is made that the foreground affected by the illumination change is similar to the texture of the background, which is used to detect the occurrence of illumination changes. The history of the background is saved so that the matching algorithm works robustly against the changes. By restoring the previous background, this works well when there is no significant change in the background before and after the illumination change occurs temporarily—still, they cannot handle the persistent change. In the paper, it is also mentioned that if the changes occur after the object is abandoned and before the event alarm occurs, the tracking can fail due to the loss of existing foreground information.

The dual background model [10,11] consists of a short-term model with a fast learning rate and a long-term model with a slow learning rate. Stationary objects are extracted from the difference between these two models. One of the disadvantages is the high false-positive rate resulting from the incompleteness of the background model. There is also a lack of consideration for the continuity of the images. Several follow-up studies have been conducted by K. Lin et al. [6,12], who created the abandoned object dataset (ABODA). Their study tried to improve the performance of abandonment detection by analyzing the pixel state of the continuous long-term and short-term foreground images for a sufficient time using a pixel-based finite state machine (PFSM). Their method involved tracking the pixel state of the foreground images until the timer expires and determining if the group of pixels maintained until this time is an abandoned object. One of the problems with these techniques is that tracking becomes complicated when another object occludes the target object. Another problem is that the background model gradually absorbs the foreground into the background. Once the absorption terminates, the tracking is impossible, which leads to poor tracking accuracy. Also, the techniques did not work robustly in their illumination change scenarios. Wahyono et al. [13] and A. Filonenko et al. [14] used the difference between the reference background model and the current background model, rather than using the short-term and long-term models. Their study used the adaptive background modeling technique to withstand the temporary illumination changes but failed in ABODA’s large, persistent illumination change scenarios. D. Shyam et al. [15] extract stationary objects from the dual background model using the sViBe [16] background modeling method, which integrates color-based ViBe proposed by Barnich et al. [17] and texture-based SILTP proposed by Liao et al. [18]. They also used a pixel-based FSM to track and the deep learning method SSD (Single Shot MultiBox Detector) to detect humans and suspicious objects. They showed excellent performance on the ABODA’s illumination change scenarios. However, their algorithm is also highly dependent on the foreground information generated from the background model like others. Therefore, it seems that they are unlikely to work robustly against illumination changes that occur after an object is unattended and before the event alarm is triggered. C. Cuevas et al. [19] suggest a triple background model consisting of three short-term, mid-term, and long-term models to make it possible to track an object for a long period. In this model, the short-term model absorbs foreground faster than the mid-term model. The long-term model never absorbs the stationary foreground objects into the background regardless of how long they have not been moved. The model can track a stationary object for a long time, and occlusion is also easily handled. However, the model cannot adequately accommodate illumination changes. If the changes happen, the affected new foreground images remain unabsorbed in the long-term model, generating a large number of wrong stationary objects.

Deep learning related studies are also in progress. S. Smeureanu et al. [20] use a two-stage approach, which detects all stationary objects in the first stage and selects abandoned ones from the detected objects in the second stage. By training the image of the person with an object as a negative image, they consider all objects near the person to be attended. They also create composite images of various luggage images placed at random locations in the measured background and train the detection model by labeling it as a positive image. This method is concerned with overfitting in terms of deep learning techniques, and in scenarios, there seems to be no consideration of owner detection and illumination changes. S. V. Sidyakin et al. [21] train their detection model using various luggage images, and at the same time, the detailed images of objects in a specific background labeled as the background images. As above, an overfitting problem may occur that only works well with a particular background learned previously.

## 3. Robust Detection of Abandoned Objects in Illumination Changes

### 3.1. General Detection Process

In this paper, the proposed algorithm detects abandoned objects using the PETS2006 abandoned object criteria [5,12]. The requirements for PETS2006 abandoned object detection are as follows:

- Temporal rule: The luggage is declared an un-attended object when the owner leaves it and disappears, and it is not re-attended within time T = *n* seconds.
- Spatial rule: When the distance between the owner and the luggage is longer than a pre-defined distance, then an alarm event is triggered.

**Figure 1:**
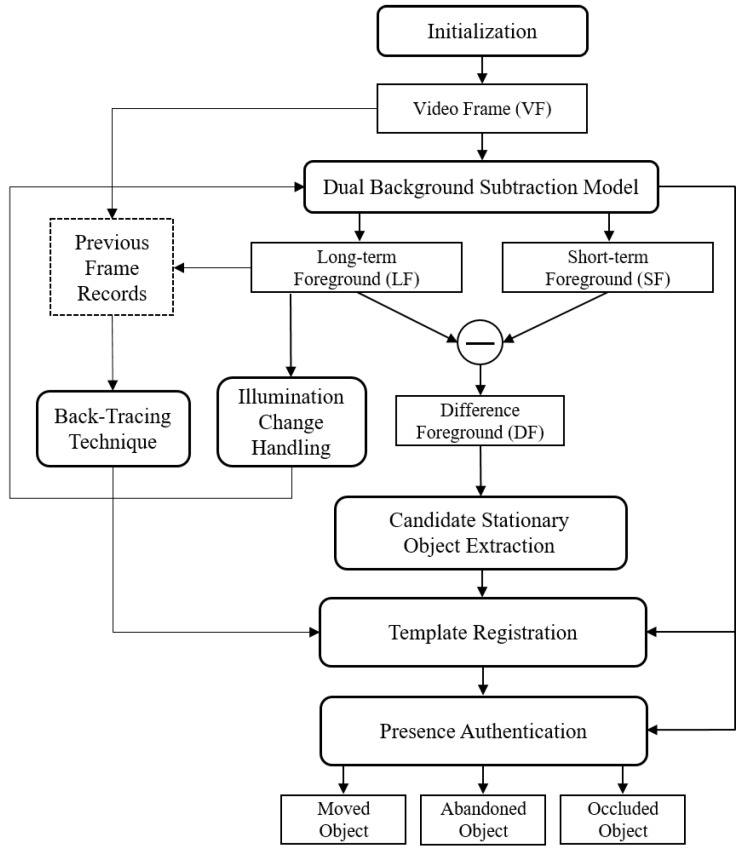
Framework of the proposed algorithm.

Figure 1 shows the framework of the proposed algorithm. The dual background model, which consists of two background models with different learning rates or absorption rates, generates a long-term foreground (LF) and a short-term foreground (SF) from a single video frame (VF) image. Since the long-term model learns slower than the short-term model, stationary foreground objects remain for a relatively long time in the LF, except for moving objects that exist equally in both models. The difference foreground (DF) is generated by simply subtracting the SF from the LF to extract only stationary foreground objects. The proposed algorithm saves the previous frame records, including the original video frame records and the long-term background (LB) records generated from the long-term model. They are used for the back-tracing technique to find the owner of an abandoned object and to obtain the contours of a background image.

In the DF, foreground objects which have a blob size smaller than a certain threshold are considered as noise and filtered out. The objects that pass the filtering process can be abandoned objects that we need to track, temporarily stationary people, or small light spots. For selecting a candidate stationary object among those objects, the proposed algorithm first checks whether the foreground object of the same size repeatedly appears at the same location in the DF to filter out the effects of temporarily stationary objects or small light changes. In this paper, we call this the object stability verification, which is deployed to overcome the incompleteness of the dual background model. If a stationary foreground object appears at the same location for a specified period, which is sufficiently small compared with the whole tracking time, the object is considered stable. If not, it is discarded. Once the stability of a stationary foreground object is verified, the algorithm determines whether it is a standing person, which occupies a large percentage of the various causes of false alarms in the surveillance system [7]. If the object is a human, it is also discarded. Otherwise, it becomes a candidate stationary object. At this time, the algorithm creates a template of the candidate stationary object to track. When the owner of the object leaves, the timer for abandonment determination starts, and the algorithm waits until the end of the timer without any visual analysis related to the object tracking. If the timer expires, the presence authentication process is performed to verify the existence of the candidate stationary object in the current video frame, which determines the next or final state of the object such as abandoned, occluded, and moved. In this paper, we defer the object state determination until the abandonment determination time (*n* seconds). However, if necessary, it is possible to grasp the state every second by arbitrarily modifying the *n* value. The important fact is that once the template is registered, there is no need to analyze the foreground of the candidate stationary object until the timer elapses. Considering that most of the existing studies have suffered from the problem of the disappearing foregrounds over time, the proposed methods can be a breakthrough to solve this problem.

### 3.2. Illumination Change Handling

To handle illumination changes, a technique that quickly detects and adapts to illumination changes while continuing to track the objects is necessary. We deployed the following three methods to handle the illumination changes:

1. Rapid detection and adaptation of illumination changes
2. Template registration and presence authentication for candidate stationary objects
3. Object comparison based on the largest contour

The pseudo-code below shows the illumination change adaptation method proposed in this paper.

Illumination Change Handling:
    
if (The size of blob[*i*] in LF ≥ *Th*)
then /*An illumination change is detected*/
  Short-term-model→learning-rate(maximum);
  Long-term-model→learning-rate(maximum);
  Illumination_change_flag = true;
    
if (The number of blobs in DF == 0 && Illumination_change_flag == true)
then /*The adaptation is terminated*/
  Short-term-model→learning_rate(original_short-term-learning-rate);
  Long-term-model→learning_rate(original_long-term-learning-rate);
  Illumination_change_flag = false;

The proposed algorithm considers that illumination changes occur simply when a blob larger than a specific threshold value (*Th*) appears in the LF. Then, it immediately increases the learning rate of each model equally, forcing both models to adapt quickly to the circumstance at the same time as soon as the occurrence of an illumination change is detected. Afterward, we need to find out whether adaptation terminates. The algorithm determines that both foregrounds of each model become completely identical if there is no blob detected in the DF image. In other words, they perfectly adjust to the changes after absorbing all of the affected parts into the background. And then, both learning rates return to their original values. The illumination change handling technique can quickly detect and adapt to the changes. However, it also has the limitation that existing meaningful foreground information could disappear after adaptation is over because the algorithm reinitializes the learning rates of both models. So, this paper suggests the template registration and presence authentication are used to solve this problem.

When a candidate stationary object is selected, a template of the object is created and registered. The template consists of the position, size of the object, the largest contour of the object image, the largest contour of the background image, and the color histogram of the owner. The position and size of the object are necessary to set the region of interest for further tracking. The largest contour of the object image is going to be used in the next presence authentication, which determines whether a candidate stationary object still exists in the current frame. As mentioned above, the system proposed in this paper saves and manages the previous frame records, including the original video frames and the background records for the back-tracing technique that performs background image extraction and owner detection. The largest contour of the background image is also required for presence authentication. It can be obtained from the previous long-term background image right before the object is revealed as foreground in the LF and used to determine whether the object is occluded or moved in the presence authentication. The owner can be found in the previous frame record of the frame where the object first appeared in the foreground and used to check whether the object is unattended to start the timer and also the re-attendance of the owner in video scenarios.

Once the template is registered, the algorithm keeps checking whether the owner leaves the object. When the object is unattended, the timer for abandonment determination is started, and the proposed algorithm does not perform video analysis for tracking on the region of interest until the pre-defined *n* seconds has passed, that is, the end of the timer, which is quite different from most existing studies that consistently perform the foreground analysis for tracking. Also, we don’t need to use general tracking methods such as the optical flow [22] or Kalman filters [4,23] because we are tracking stationary objects rather than moving objects. If the owner appears near the object again, the tracking is aborted—unless, after the timer expires, the algorithm performs the presence authentication. The pseudo-code of the presence authentication for a candidate stationary object (CSO) is as follows:

Presence Authentication:
    
if ((matching_score(CSO.theLargestObjectContour, theCurrentLargestContour of CSO.area) ≥ *Th*)
then /*abandoned object*/
    alarm AbandonedObjectDetection;
else
  if ((matching_score(CSO.theLargestBackgroundContour, theCurrentLargestContour of CSO.area) ≥ *Th*)
  then /*moved object*/
      discard CSO;
  else /*occluded*/
      repeat Presence Authenticaion after pre-determined time passes;

In the beginning, the presence authentication performs by comparing the largest contour of the current object image with that of the candidate stationary object in the registered template. If those are identical, the algorithm logically assumes that the object has been stationary for *n* seconds, and it is determined as an abandoned object. Otherwise, the algorithm compares the two largest contours of the current object image and the background image in the template. If they are equal, which means that the current image is the same as the background, the object should be considered to have disappeared in the middle of tracking. If not, the algorithm determines that another object occludes the object at present. For the occluded objects, the presence authentication is repeated after pre-determined time passes to determine the final state of the object.

The reason for using the largest contour, rather than others such as the whole contours and color histogram, is that it is more robust to illumination changes according to our experiments. When illumination changes occur, there could be small or massive differences in the detail of the object. For example, the direction and shape of several shadows, and the color of the object can change, which makes the system mistakenly determine the objects of before and after the changes are different. Color histogram comparison, a simple way to calculate the similarity of two images, works well under normal circumstances but is very vulnerable to illumination changes. Therefore, we need something less susceptible to illumination changes to compare object images. For this, we use the texture information of the object. Contour, an element of edges, has the advantage of measuring and utilizing specific areas because it is the closed curve. The contours of the object area contain the entire object and part of the background. As the light changes, the direction and size of the shadow can change, which affects the internal details of the object. Therefore, the contours inside the object are relatively vulnerable to illumination changes. That is why we use the largest contour for the template registration and presence authentication. This paper uses the findContours and matchShapes function of OpenCV for comparing with contours in experiments [24]. The findContours retrieves contours from the binary image using the algorithm proposed by Suzuki et al. [25]. The matchShapes function compares two shapes and uses the Hu invariants as follows:

(1) $$I\left(A,\;B\right)=\sum_{i=1\ldots 7}\left|\frac{1}{m_{i}^{A}}-\frac{1}{m_{i}^{B}}\right|\;(\mathrm{where}\;m_{i}^{A}=\;\sin h_{i}^{A}\cdot \log h_{i}^{A},\;\;m_{i}^{B}=\;\sin h_{i}^{B}\cdot \log h_{i}^{B})$$

where A denotes contour1, B denotes contour2, and $h_{i}^{A},\;h_{i}^{B}$ are the Hu moments of A and B, respectively. If the return value of the function is 0, the two contours are exactly the same, and the closer to 0, the higher the similarity.

In summary, this paper proposes an illumination change handling method that reduces the false alarm rate by quickly adapting to the sudden change, which is generally generating many foreground blobs with various sizes, so that the wrong foreground object in the DF is not selected as a candidate stationary object. Besides, the template registration of candidate stationary objects and the presence authentication using the largest contour enable robust tracking and accurate abandonment determination, even though the illumination change occurs during the object tracking.

## 4. Experiments

We used the k-nearest neighbor (KNN) [26] background subtraction technique as a background modeling method and the histogram of oriented gradients (HOG) [27] as a human detector. In our algorithm, the foreground of a stationary object only needs to be maintained until its stability is verified, i.e., for a relatively small amount of time, which simplifies the configuration of the learning rates so that their difference is just sufficiently large. In our experiments, the learning rate ratio for the short-term and long-term models is 50:500. For the performance evaluation, we tested our algorithm using the popular datasets PETS2006 [5] and ABODA [28]. ABODA’s video 6, 7, and 8 are scenarios to verify that the proposed algorithm is robust to illumination changes. However, since they only deal with illumination changes that occur before objects are abandoned, we have further experimented with our video dataset that covers the situation of illumination changes occurring after tracking starts and before the timer for the abandonment determination ends.

### 4.1. PETS2006

The PETS2006 dataset consists of seven scenarios in an indoor environment. Each scenario addresses issues related to the detection of abandoned objects in normal circumstances, such as stationary people, bags as large as people, situations where a person drops and retakes luggage, tracking an attended object for a long time, and access from non-owners. However, they don’t deal with the illumination change issue.

**Figure 2:**
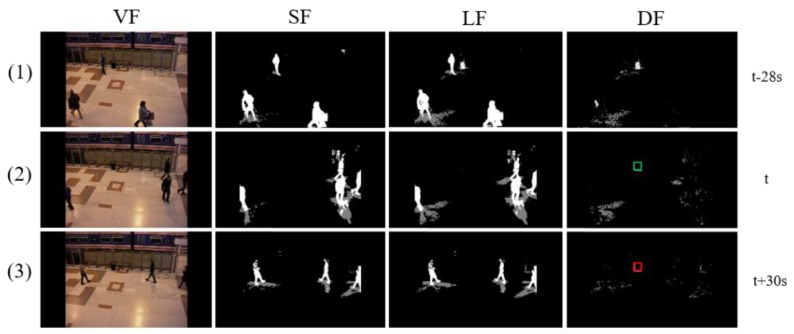
Abandoned object detection in PETS2006 Scenario 7. (VF, SF, LF, and DF represent video frame, shot-term foreground, long-term foreground, and difference foreground, respectively).

Figure 2 shows the detection process of an abandoned object in Scenario 7, which has the greatest difficulty in the PETS2006 dataset. The owner is loitering around an object for quite a long time and disappears. Several groups of people then pass near the object. One of the significant issues in this scenario is the long-term tracking of a target object because the foreground object is gradually absorbed into the background. Another issue would be the ability to distinguish the owner with others. We can see that the foreground of the target object has already disappeared, and the owner leaves the object at (2). But the algorithm still can track the object and it finally determines the object as an abandoned object by the presence authentication at (3).

Table 1 shows the test results of the proposed algorithm for the PETS2006 dataset. GT, TP, FP, and FN mean ground truth, true positive, false positive, and false negative, respectively. As we can see, all scenarios have 100% precision. In Table 1, the Owner means the number of owners of abandoned objects. The proposed algorithm uses back-tracing techniques to detect owners in all scenarios except Scenario 3, where a person drops an object and then takes it back. The algorithm uses the owner information for checking the owner’s re-attendance that often occurs in a real environment, though the detail is not covered in this paper.

### 4.2. ABODA

ABODA is the dataset created by K. Lin et al. [6,12,28]. Eleven scenarios are presented, covering indoor, outdoor, night, illumination changes, and crowded scenes. Since ABODA does not follow the temporal standard of PETS2006, which is 30 s, we arbitrarily lowered the abandonment determination time for the experiment. We excluded video 11, which does not comply with the PETS2006 abandonment rules from both temporal and spatial viewpoints.

Table 2 shows the experimental results of our algorithm and several previous studies on the ABODA dataset. In particular, the results for video 6, 7 and 8, which are related to illumination changes, show that the algorithms proposed in other papers did not present satisfactory results except for D. Shyam et al. [15]. However, they don’t cover the issue of illumination changes occurring between the start time and the end time of tracking, which is discussed in the next section. The proposed algorithm also detects abandoned objects exactly in all scenarios, which is the result of illumination change handling techniques that can quickly determine the occurrence of illumination changes and adapt to them.

**Figure 3:**
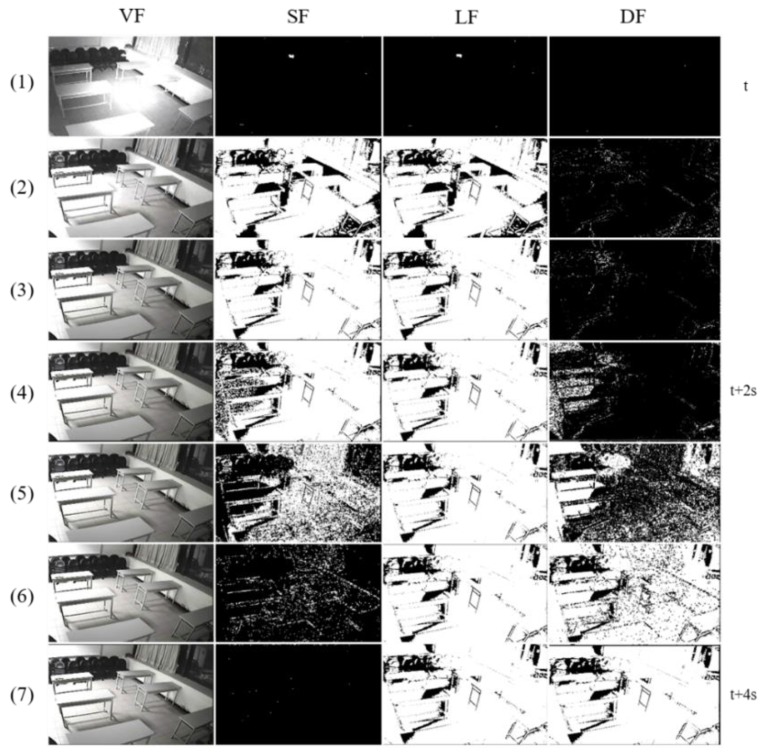
Illumination changes in ABODA video 7 without our illumination change adaptation technique.

Figure 3 shows how the proposed algorithm works for ABODA video 7 without our illumination change handling technique. We can see that when illumination changes occur, many foregrounds of various sizes are generated in the SF and LF images. The short-term model, which has a high learning rate, adapts quickly to the changes, but the long-term model is continuously affected by the changes for a long time. From the end of the short-term model’s adaptation, many stationary foreground objects are created in the DF, which makes the algorithm select wrong candidate stationary objects significantly increasing false detection rates.

**Figure 4:**
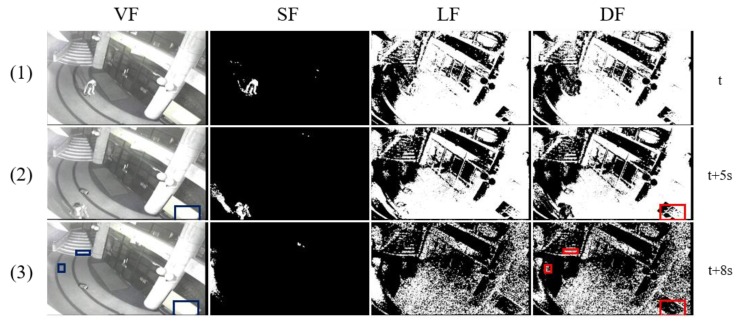
This shows the test result for ABODA video 6 without illumination change adaptation technique.

In Figure 4, even though the filtering mechanism of our algorithm can filter small noise, stationary human objects, and objects without owners, we can see that several wrong objects marked by the red rectangle are extracted as candidate stationary objects in the DF at (2) and (3) due to the effect of an illumination change. It shows why the technique for the rapid detection and adaptation of illumination changes is necessary for video surveillance systems.

**Figure 5:**
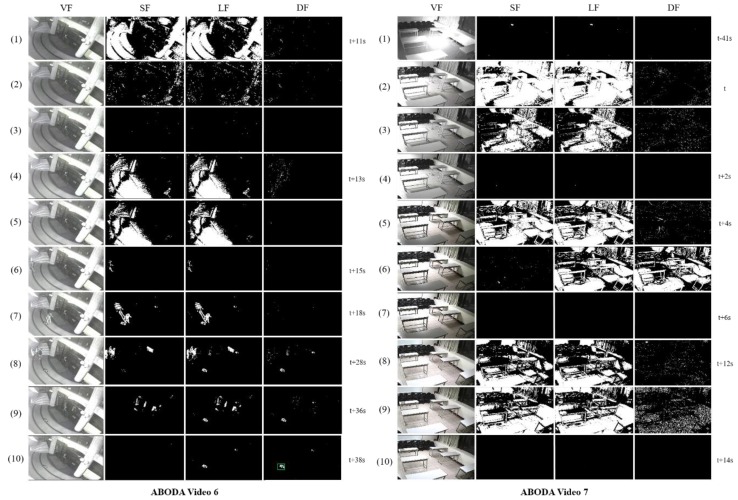
Illumination change handling in ABODA video 6 and video 7.

Figure 5 shows the adaptation process of the proposed algorithm for ABODA video 6 and video 7 with the illumination change handling method. We can see that it quickly detects and starts the adaptation process for all illumination changes at (1) and (4) of video 6 and (2), (5), and (8) of video 7, respectively. Each adaptation takes less than two seconds in the experiments, which can be regarded as small enough compared to the total tracking time for the abandonment determination. In video 6, a person appearing after the adaptation of an illumination change abandons an object and the proposed algorithm selects the object as a candidate stationary object at (10).

**Figure 6:**
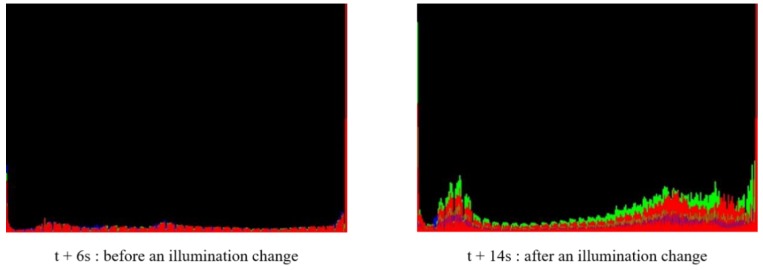
Histogram of the whole video frame image with an illumination change in ABODA video 7.

Figure 6 shows the entire image color histogram at (7) and (10) of Figure 5 before and after light changes. We can see that light changes affect the entire image significantly, which can increase the false alarm rates or alter the characteristics of the target object. The illumination change handling method should handle both of them to detect abandoned objects robustly.

**Figure 7:**
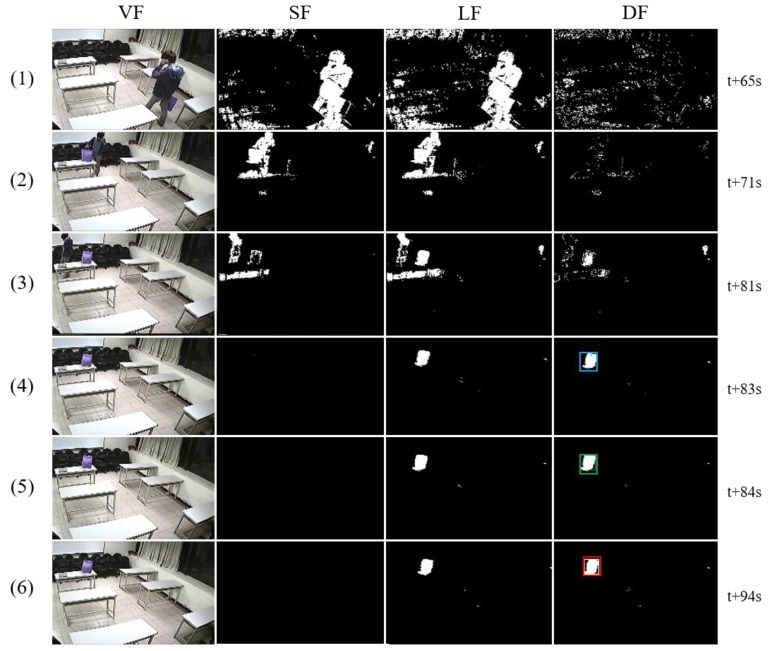
Abandoned object detection in ABODA video 7.

Figure 7 covers the situation of abandonment after illumination changes in ABODA video 7. Thanks to the illumination change adaptation mechanism of this paper, it shows that there is no effect of the changes that happened earlier. The algorithm accurately detects only the abandoned object even though an illumination change occurred before. The DF in (5) shows a candidate stationary object that is stable, non-human, and unattended. At this point, the template of the object is created. Since the largest contour of the current video frame is the same as the largest contour of the object in the template, the object is determined as an abandoned object through the presence-authentication process in (6). 

Table 3 shows the image, color histogram, contours, and the largest contour of the tracking object at (5) and (6) of Figure 7. We can see that the object remains the same since there was no light change from the time when the candidate stationary object was selected until the timer expired. As a result, the histograms of the two images are almost the same, and the shape matching result using the largest contour among the extracted multiple contours is very close to zero, that is, the two objects are equal. This means that there is no big difference in object comparison based on histogram and the largest contour in normal circumstances. However, the situation would be totally different if an illumination change were to occur during the object tracking, which is discussed in the next section, experimented by using our dataset.

### 4.3. Our Dataset

We made two video scenarios, KICV (Koreatech illumination change video) 1 and 2, to measure the robustness against the illumination changes that occur after abandonment behavior in indoor and outdoor environments, respectively.

**Figure 8:**
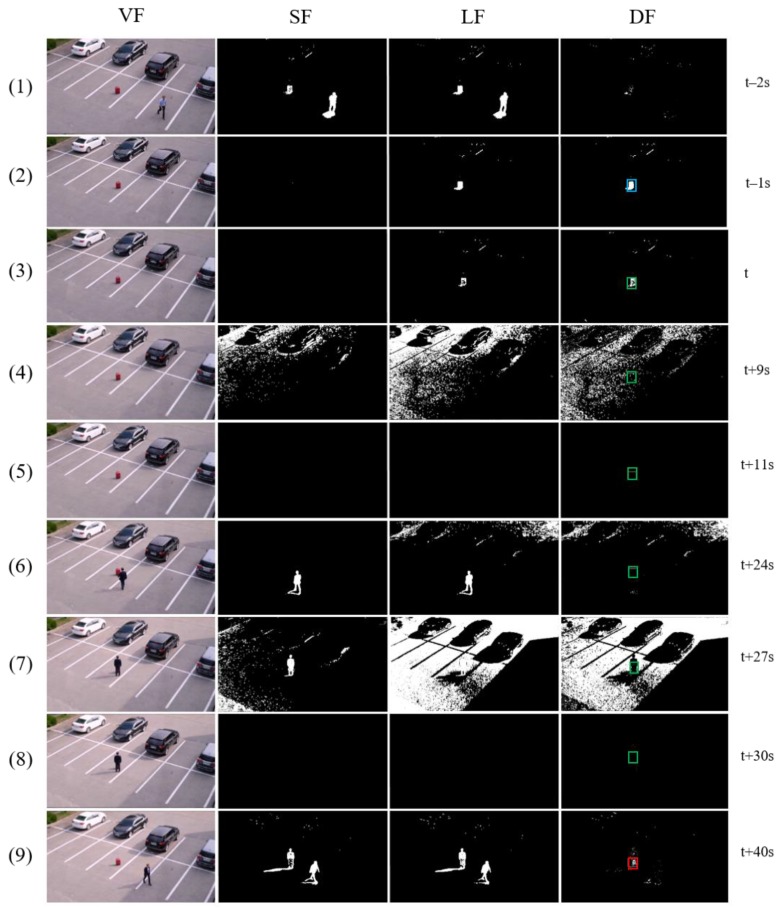
Outdoor illumination changes in KICV (Koreatech illumination change video) video 1.

KICV video 1 deals with complicated issues, including illumination changes, owner’s re-attendance, occlusion, and long-term abandonment that occur in the middle of tracking an abandoned object in an outdoor environment. In Figure 8, there are two illumination changes after a candidate stationary object is selected at (3). At this time, the largest contour of the candidate stationary object and the largest contour of the background image are extracted and registered together as attributes of a template. Although the foreground object is quickly absorbed into the background through the following illumination change adaptation, we can see that the algorithm still can track the object robustly. A person is approaching the object at (6). So, the algorithm checks whether the person is the owner using the owner histogram of the template and determines that he is not the owner. Then, it does not stop the timer. At (8), the figure shows the situation where the predefined abandonment time *n* seconds (*n* = 30 in the experiment) have passed since the object was unattended. The presence authentication is executed, and it compares the largest contour of the current object image with that of the candidate stationary object in the template. Because a non-owner currently occludes the object, the result fails. Then, the largest contour of the current object image is compared with that of the background image in the template. Because they do not match each other, the state of the object is determined to be an occluded object in the presence authentication process. The proposed algorithm performs the presence authentication again after a particular time (10 s here) in this case. We can see that when the person disappears, the object is determined to be abandoned at (9). The largest contour of the current image equals that of the candidate stationary object in the template, thus, the presence authentication succeeds.

**Figure 9:**
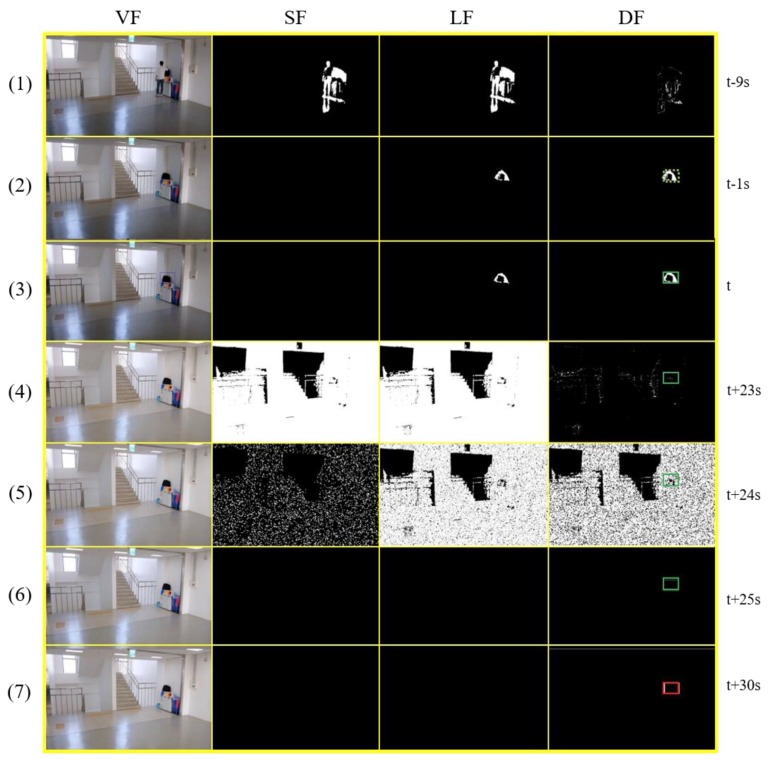
Indoor illumination changes in KICV video 2.

The KICV video 2 deals with an indoor situation. In Figure 9, a candidate stationary object is determined and the template of this object is registered at (3). After that, an illumination change occurs before abandonment determination at (4), but the proposed algorithm correctly detects the abandoned object at (7) by using the presence authentication based on the largest contours, although the shape of the current object is slightly different from the previous image.

In Table 4, the color histogram changes significantly as the tracking object is affected by light changes. As shown in Table 3, the histogram matching score is 97.8% in the case of no illumination change during object tracking. However, although the object after light changes seems almost the same as before, the histogram matching score drops below 30% in Figure 8. Therefore, there is a limit in the color-based comparison. On the other hand, the results of the shape matching using the largest contour are relatively very positive in the same situation. Other than when occlusion occurred in KICV video 1 (t + 30 s), the result value is still close to zero, although the final shape of the object in both videos changed slightly by the illumination change. In this way, the proposed algorithm can track the object stably robust to light changes and determine whether the object is abandoned by using texture information, not color, especially the largest contour.

### 4.4. Challenging Issues

The verification of an owner’s re-attendance is quite a challenging issue in the detection of an abandoned object. To check the owner’s re-attendance, we used the color histogram information of the owner registered as an attribute of the template. If the timer has not expired yet, it is compared with the histogram of a human approaching near the object, which performs well in our experiments because there is no scenario where the histogram of the owner is changed by illumination changes. However, if such a case happens, the accuracy of a histogram-based owner comparison would be getting lower, because the histogram is largely affected by the changes, as we learned in the experiments. Therefore, it needs to be enhanced to be more robust in illumination changes. Additionally, we will cover experiments to see the limitations of color and texture information using methods such as blurring filters.

## 5. Conclusions

In this paper, we presented an algorithm for detecting abandoned objects robustly in illumination changes as well as other complex circumstances like occlusion, long-term abandonment, and owner re-attendance. The algorithm is based on a dual background model, which is integrated with the illumination change adaptation mechanism and the largest contour-based presence authentication mechanism.

The proposed algorithm can quickly detect various illumination changes and adapt to them immediately, which avoids generating wrong stationary objects related to poor performance. It is also possible to track the target objects even if the foreground information disappears caused by the nature of the background subtraction technique or the occurrence of illumination changes, by using the template registration and presence authentication. When a candidate stationary object is selected, a template of the object is created and registered. The template consists of the position, size of the object, the largest contour of the object image, the largest contour of the background image, and the color histogram of the owner. When the timer for abandonment decision expires, the presence authentication is executed by comparing the largest contour of the region of interest in the current video frame with that of the object or background in the registered template, which determines whether the candidate stationary object is an abandoned object or not. Since the largest contour is rarely affected by illumination changes, the proposed presence authentication works well even in the illumination changes that occurred in the middle of tracking as well as normal circumstances. We have tested the algorithm on the PETS2006 dataset to demonstrate the ability to detect abandoned objects under normal circumstances. We also proved that it is robust even in the more diverse environments of the ABODA dataset. In particular, it shows that the illumination change handling mechanism works well in practice. However, since the ABODA’s illumination change scenarios don’t deal with the light changes that occur during object tracking, we performed additional experiments on our videos. In the experiments, we demonstrated that even when light changes occur during tracking, the external shape of the object being tracked, that is, the largest contour, is less affected, which enables the presence authentication to be performed properly. Our future work will be focused on the issue of verifying the owner’s re-attendance in illumination changes. We will also cover experiments to see the limitations of color and texture information using methods such as blurring filters.

## Figures and Tables

**Table 1:** Test results for PETS2006 dataset.

Video	GT	TP	FP	FN	Owner
S1	1	1	0	0	1
S2	1	1	0	0	1
S3	1	1	0	0	-
S4	1	1	0	0	1
S5	1	1	0	0	1
S6	1	1	0	0	1
S7	1	1	0	0	1

**Table 2:** Comparison of test results for ABODA (abandoned object dataset).

Video	Scenario	GT	Proposed		Lin [6]		Wahyono [13]		Ilias [29]		Patrick [30]		Wentong [31]		Shyam [15]	
			TP	FP	TP	FP	TP	FP	TP	FP	TP	FP	TP	FP	TP	FP
**V1**	**Outdoor**	1	1	0	1	0	1	0	1	0	1	0	1	0	1	0
V2	Outdoor	1	1	0	1	0	0	1	1	0	1	0	1	0	1	0
V3	Outdoor	1	1	0	1	0	0	1	1	0	1	0	1	0	1	0
V4	Outdoor	1	1	0	1	0	0	1	1	0	1	0	1	0	1	0
V5	Night	1	1	0	1	1	1	0	1	0	1	0	1	0	1	0
**V6**	**Illumination Change**	**2**	**2**	**0**	**2**	**0**	**-**	**-**	**2**	**0**	**1**	**0**	**2**	**1**	**2**	**0**
**V7**	**Illumination Change**	**1**	**1**	**0**	**1**	**1**	**-**	**-**	**1**	**2**	**1**	**1**	**1**	**0**	**1**	**0**
**V8**	**Illumination Change**	**1**	**1**	**0**	**1**	**1**	**-**	**-**	**1**	**2**	**1**	**0**	**1**	**1**	**1**	**0**
V9	Indoor	1	1	0	1	0	1	0	1	0	1	0	1	0	1	0
V10	Indoor	1	1	0	1	0	1	0	1	0	1	0	1	1	1	0
V11	Crowded Scene	1	-	-	1	3	-	-	0	1	1	0	1	1	-	-

**Table 3:** Comparison results using the color histogram and the largest contour in ABODA video 7.

Object	Image	Color Histogram	Contours	Largest Contour	compareHist( ) Function	matchShapes( ) Function
Candidate Stationary Object (t)	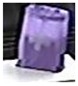	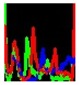	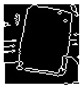	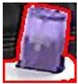	Matching Score: 97.8% (The higher, the better)	Matching Score: 0.004 (The lower, the better)
Current Object (t+10s)	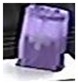	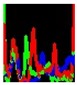	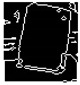	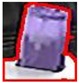		

**Table 4:** Comparison results using the color histogram and the largest contour in KCVI dataset.

Video	Object	Image	Color Histogram	compareHist( ) Function	Largest Contour	matchShapes( ) Function
KICV Video 1	Candidate Stationary Object (t)	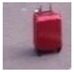	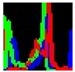	Matching Score: 22.4% Matching Score: 29.0%	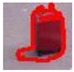	Matching Score: 0.540 Matching Score: 0.059
	Current Object (t+30s)	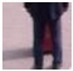	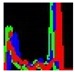		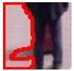	
	Current Object (t+40s)	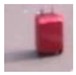	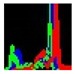		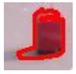	
KICV Video 2	Candidate Stationary Object (t)	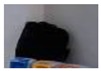	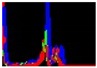	Matching Score: 9.6%	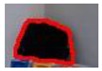	Matching Score: 0.057
	Current Object (t+30s)	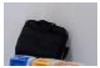	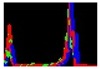		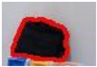	
